# Software-Defined Optimal Computation Task Scheduling in Vehicular Edge Networking †

**DOI:** 10.3390/s21030955

**Published:** 2021-02-01

**Authors:** Zhiyuan Li, Ershuai Peng

**Affiliations:** 1 College of Computer Science and Communication Engineering, Jiangsu University, Zhenjiang 212013, China; 2221808035@stmail.ujs.edu.cn; 2 Jiangsu Province Big Data Ubiquitous Perception and Intelligent Agricultural Application Engineering Research Center, Zhenjiang 212013, China; 3 Jiangsu Key Laboratory of Security Technology for Industrial Cyberspace, Zhenjiang 212013, China

**Keywords:** software-defined vehicular edge networking, resource allocation, computation task scheduling, optimal control

## Abstract

With the development of smart vehicles and various vehicular applications, Vehicular Edge Computing (VEC) paradigm has attracted from academic and industry. Compared with the cloud computing platform, VEC has several new features, such as the higher network bandwidth and the lower transmission delay. Recently, vehicular computation-intensive task offloading has become a new research field for the vehicular edge computing networks. However, dynamic network topology and the bursty computation tasks offloading, which causes to the computation load unbalancing for the VEC networking. To solve this issue, this paper proposed an optimal control-based computing task scheduling algorithm. Then, we introduce software defined networking/OpenFlow framework to build a software-defined vehicular edge networking structure. The proposed algorithm can obtain global optimum results and achieve the load-balancing by the virtue of the global load status information. Besides, the proposed algorithm has strong adaptiveness in dynamic network environments by automatic parameter tuning. Experimental results show that the proposed algorithm can effectively improve the utilization of computation resources and meet the requirements of computation and transmission delay for various vehicular tasks.

## 1. Introduction

With the development of the intelligent connected vehicles, more and more vehicular terminal devices have begun to participate in data task collection and processing [1,2,3,4], such as autonomous driving, virtual reality, and computation-intensive tasks. [5] presented a traffic management service, called ABATIS, to optimize the routes for vehicles. In cloud computing paradigm, the collected data and processing tasks have been uploaded to a centralized data center far away from Internet of vehicles [6]. After the task execution is completed, the processing results will be transmitted back to the task initiator. Hence, enormous sensed data and the encapsulated computation-intensive tasks transmission have put severe strain on the mobile network, especially in terms of bandwidth consumption. With the ever-increasing amount of network traffic, the network congestion frequently happens and degrades the quality of service (QoS) for delay-sensitive tasks [7,8,9].

**Figure 1:**
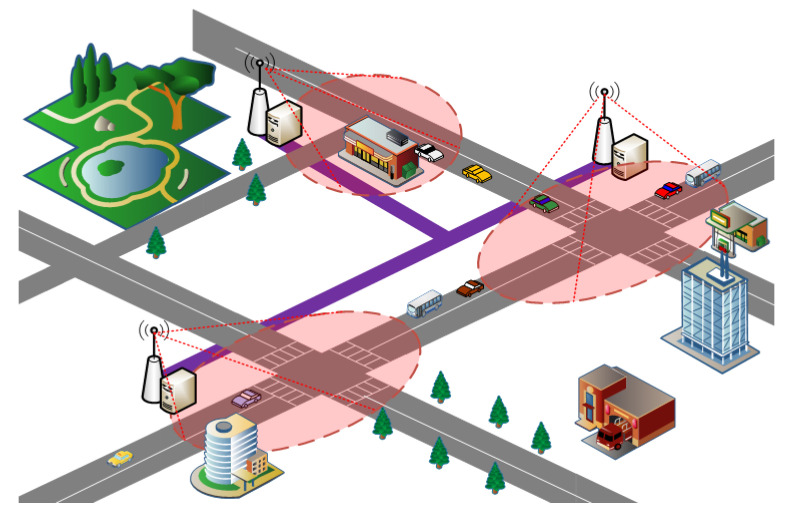
Vehicular edge computing paradigm.

**Figure 2:**
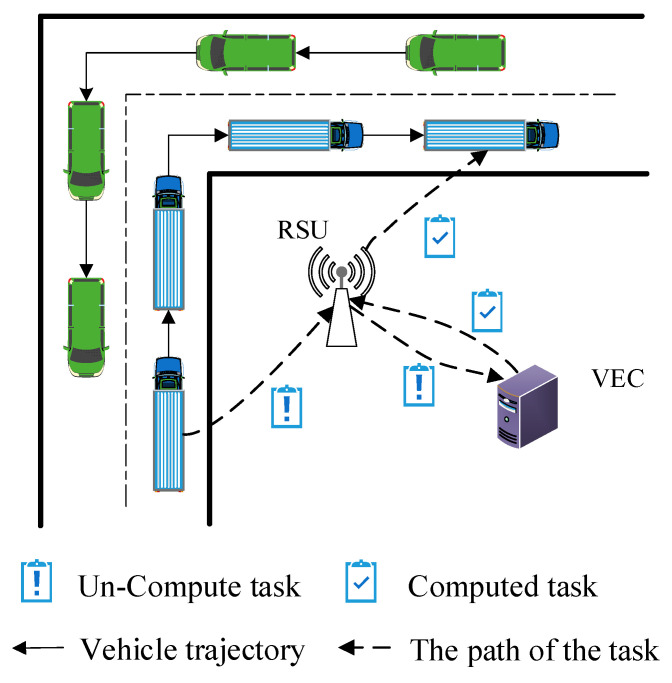
Illustration of Vehicular Edge Computing (VEC) network application scenario.

Recently, a Vehicular Edge Computing (VEC) paradigm was presented, as shown in Figure 1. A vehicle can offload some complex delay-sensitive tasks to edge cloud due to the limited computing, storage, communication capacity [8,10,11], as shown in Figure 2. There are existing works focusing on the task offloading and resource allocation. For example, Chen [12] exploits Lyapunov optimization theory to maximize the long-term performance of networking system, such as lower task execution delay and task migration delay. Although Lyapunov optimization algorithm can be designed and implemented, it is not suitable to complex and highly dynamic network environments. Literature [13] proposed the Cloud Computing Management Unit (CCMU) to optimize the allocation of computing resources. In [13], CCMU uses the Markov decision process to give the optimization decisions. Although the MDP has been developed a general algorithm (Bellman Equation), the convergence speed of MDP is still slow for the state space of continuous variables. Overall, it is difficult to achieve the load balancing effect for the existing task offloading and resource allocation algorithms. How to reasonably allocate computing and communication resources is still a challenge for the VEC networking. In our previous research, our work is the exploiting the modern control theory to model the computation task optimal scheduling in vehicular edge networks [14]. This paper is the extension of the conference paper [14]. In this paper, we proposed an Optimal Control-based computing Task Scheduling algorithm (OCTS). The proposed OCTS method has significantly improved the previous work in the following aspects: (1) Introducing VEC architecture and assumptions; (2) using the software-defined network (SDN)/OpenFlow to collect the necessary network parameters for task scheduling; (3) optimizing task migration delay and avoiding network congestion by adjusting the weight value ξ. When the network delay is higher than the threshold value, it can be reduced by increasing weight value ξ; (4) enhancing the experimental verification; and (5) comparisons with the existing approaches.

The remainder of this paper is organized as follows. Section 2 gives the related work. Section 3 describes the network scenario and system model. Section 4 presents the vehicular task queuing model. Section 5 proposes the optimal control-based computing task scheduling algorithm. Section 6 presents our empirical studies. Section 7 concludes this paper.

## 2. Related Works

With the development of Internet of vehicles, more and more vehicular applications have been developed to meet the QoS requirements of mobile users. Due to the limited computing, storage, and communication capacities of mobile terminals, the vehicle itself cannot be able to meet the QoS requirements of various applications. Although cloud computing has large computing power, the transmission delay between vehicles and cloud computing platform is significant [15]. Hence, it is not sufficient for the latency-sensitive applications. The edge computing servers are closer to the mobile terminals, which can effectively reduce task execution and migration delay [8].

Nowadays, some researchers have focused on the mobile task offloading and resource allocation. A previous study [16] proposed a joint optimal VEC server selection and offloading (JSCO) Algorithm to address resource allocation for a multiuser multi-server VEC system. Fan [17] proposed a collaborative optimization migration and caching model to improve the performance of edge task execution. Then, the optimization problem with two independent sub-problems was solved. Next, the resource management algorithm was designed to jointly schedule task and its migration. The experimental results show that the proposed model can reduce the latency of task execution. Another study [18] studied the average delay minimization problem for component-based linear applications in vehicular ad-hoc networks and proposed a delay-optimization based ant colony optimization algorithm (DoACO). This optimization problem with the stringent constraint conditions has been proved a NP hard problem. Liu [19] presented the optimization problem with the energy consumption, execution delay, and price cost constraints. Specifically, energy consumption, execution delay, and computing capacity were explicitly and jointly considered. On the basis of theoretical analysis, a multi-objective optimization problem with joint conditions was presented. To minimize the power consumption, execution delay, and price cost, the multi-objective optimization problem was solved by the scaling scheme and the interior point method. Another study [20] proposed a resource-sharing scheme for data center collaboration, which stipulated that each data center uses buffer to store service requests for local execution. When the buffer is full, the request is migrated to an adjacent data center and accepted if the current queue length is below the pre-defined threshold in the data center. In this way, the blocking state and task execution latency of the data center can be effectively reduced. Furthermore, previous research [21] studied three load sharing schemes, namely no-sharing, random sharing, and minimum load sharing. After the comparisons, they find that the minimum load sharing scheme is most suitable for making full use of the cooperation among servers to realize the load balancing. Another study [22] proposed another edge computing task scheduling model, which transformed the waiting time minimization problem into an overall planning problem, and then carried out optimal scheduling through dynamic programming. A previous study [23] proposed an improved chaotic bat swarm algorithm. Based on the bat algorithm, chaos factors and second-order oscillation were introduced to accelerate the update of dynamic parameters and thus improve the convergence of the algorithm.

However, the proposed methods [12,20,21,22,23,24,25,26,27,28] result in unbalancing resource allocation for vehicular tasks due to dynamic network environment driven by humans. In order to achieve the load-balancing, literatures [29,30] used the idea of software defined network to obtain the more network status parameters. SDN is an innovative network design, implementation, and management method, which separates network control from forwarding process to achieve better user experience [31]. Next, the collected network status parameters are the input vector of the proposed optimization model. Experiments show that the proposed algorithms can significantly reduce task execution time. Although the proposed algorithms can achieve the better results, the number of iterations being solved is still large due to the high dimension input vector. Hence, the presented solving algorithms are not fundamentally suitable for delay-sensitive vehicular task execution.

## 3. VEC Architecture and Assumptions

In this section, we first introduce the networking layer architecture. Then, the software-defined vehicular edge networking (SD-VEC) architecture and computation models are elaborated.

### 3.1. SD-VEC Networking Layer Architecture

**Figure 3:**
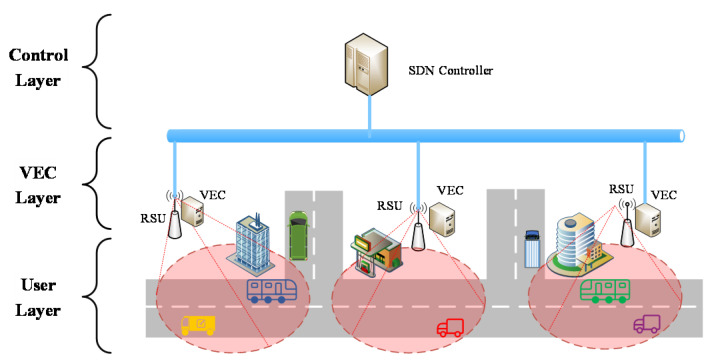
Software-defined vehicular edge networking (SD-VEC) networking layer architecture.

Figure 3 shows the SD-VEC networking layer architecture. In the user layer, when the vehicles in the area covered by a Roadside Unit (RSU), they can send vehicular tasks to the RSU with wireless connections. In the VEC layer. Each VEC server is connected with an RSU. The RSU will forward these tasks to the VEC servers and then the VEC server will execute these tasks and send them back to the requesting vehicles. The VEC servers in different area are connected by the wired cables in the same local area network. The VEC servers can send or receive tasks to or from other servers by these wired cables. In the control layer, the SDN controller can connect with the VEC servers through wired cables. The SDN controller can not only obtain the status information of servers and network, such as the CPU utilization, memory usage, but also control the task migration among the servers. The descriptions of symbols are shown in Table 1.

### 3.2. Network Model Assumptions

We assume that the second layer includes N VEC servers, which are deployed near the RSU. Each VEC server is equipped with an RSU thus the VEC servers and RSUs share the same index. When the vehicles move into the coverage of the RSUs, they can offload their computation tasks to corresponding RSUs via wireless link. The CPU frequency of a VEC server i is denoted as $f_{i}$ and the corresponding clock period is $h_{i}=1/f_{i}$. All of the CPU frequencies for VEC servers can be denoted as $f=\{f_{1},\cdots \cdots ,f_{i}\}$ and the corresponding CPU clock periods can be denoted as $h=\{h_{1},\cdots \cdots ,h_{i}\},i\in N$.

Secondly, we assume that the received tasks by server i follow a Poisson distribution. We denote $\lambda_{i}(t)$ as the number of the tasks arriving at the VEC server i at time t, and the task execution time on server i at time t is denoted as $m_{i}^{t}$. $\rho_{i}^{\lambda }(t)$ is denoted as the processing time to run tasks on a server i. All tasks arriving at the VEC servers are denoted as $\lambda (t)\in \{\lambda_{1}(t),\cdots \cdots ,\lambda_{i}(t)\},i\in N$ and their corresponding processing time can be denoted as $\rho^{\lambda }(t)=\{\rho_{1}^{\lambda }(t),\cdots \cdots ,\rho_{i}^{\lambda }(t)\},i\in N$. To simplify the computation, we assume that the task execution time is $m_{i}^{t}\times h_{i}$ for a VEC serve i. Hence, the server i can take $h_{i}\times m_{i}^{t}\times \lambda_{i}(t)$ to process the tasks at time t.

Thirdly, during the work of this system, the network delay cannot be easily calculated. In order to adjust and optimize the task schedule via the network state, we use the SDN to obtain the network state. The network delay obtained by SDN is defined as $D^{a}$, and the maximum network delay defined by a user is defined as $D_{\max}^{a}$.

## 4. Vehicular Task queuing Model

Due to the limited computation capacity of a VEC server and the burst of task arrivals, some VEC servers are busy, while others are free. The load imbalance will lead to a low computation resources utilization and extra computation time. Hence, we have to schedule tasks among VEC servers to alleviate the load imbalance.

**Figure 4:**
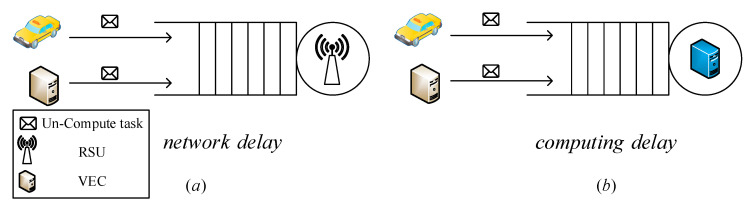
Vehicular task queuing model.

We assume that we send tasks of server i to server j and the number of the tasks can be denote as $u_{ij}(t)$. Specifically, if server i needs to process a large number of tasks at time t while server j has to process a small number of tasks, the $u_{ij}(t)$ could be positive number. Thus, the number of tasks which server i send to others can be denoted as $u_{i\cdot }(t)=\sum_{j=1}^{N_{\max}}u_{ij}(t),j\in N$. According to the presented computation model, at time *t* the VEC server *i* will take $h_{i}\times m_{i}^{t}\times (\lambda_{i}(t)-u_{i\cdot }(t))$ to process these tasks. Vehicular task queuing model is shown as Figure 4. Next, we describe the task queuing model and task computation model.

### 4.1. Task Queuing Model

Task scheduling not only can effectively decrease the task execution time of high load servers, but also can increase computation resource utilization. However, the task scheduling will increase the task transmission time of the VEC network. To calculate the task transmission time, we assume that the tasks arrive at server i from other servers follow a Poisson distribution. We also assume that the task transmission time is τ when there is no network congestion. As shown in Figure 4a, we use M/M/1 queuing model to build the vehicular task queuing model. The task transmission time can be denoted as Equation (1), where $u_{i}(t)=\sum_{j=1}^{N}u_{ji}(t)$.

(1) $$ND_{i}(u_{i}(t))=\frac{\tau }{1-\tau u_{i}(t)}$$

### 4.2. Task Computation Model

As shown in Figure 4b, we use M/M/1 to build the vehicular task queuing model. The task execution time is denoted as Equation (2).

(2) $$CD_{i}(u_{i}(t))=\frac{1}{f_{i}/m_{i}^{t}-u_{i}(t)},i\in N$$

where $CD_{i}$ is denoted as task execution time on a server i.

## 5. Problem Formulation and Optimal Task Scheduling Solving

Our goal is to reduce the load on each server as much as possible while satisfying the network delay and computing delay of vehicular tasks.

The problem formulation with constraints is shown as Formula (3).

(3) $$\begin{array}{l} \min J(u(t))=\int_{0}^{T}\left(\xi ND(u(t))+CD(u(t))\right)dt\\ s.t.\;S=\{x(T)|G(x(t))=0\}\\ \;ND_{i}(u_{\cdot t}(t))\leq ND_{\max},\forall i\in N\\ \;CD_{i}(u_{\cdot t}(t))\leq CD_{\max},\forall i\in N \end{array}$$

In the Formula (3),

(4) $$J(u(t))=\int_{0}^{T}\left(\xi ND(u(t))+CD(u(t))\right)dt$$

is the performance index of the VEC system. ND(u(t)) and CD(u(t)) is the execution time and transmission time incurred by offloaded task scheduling. When $D^{a}>D_{\max^{a}}$, the increasing value of ξ is to decrease $D^{a}$. ξ is a coefficient to adjust the weights of the offloaded task transmission time. If we increase the value of ξ, the task transmission time will decrease while the task execution time will increase. The adjustment of the ξ value is used for dealing with the stringent time constraint vehicular tasks. Formula (3) can be resolved by calculus of variations method, but before that, we need to set up the corresponding state equations and state variables.

Firstly, the CPU utilization of a VEC server is denoted as a state variable. Next, the various tasks with different QoS requirements from both vehicles and other mobile terminals (laptop, smart phones, etc.) are denoted as the inputs of the system framework. The inputs of the network system have clearly influenced on running state variables of this system. The state variables of a VEC server i can be denoted as Equation (5).

(5) $$x_{i}=\frac{h_{i}\times m_{i}^{t}\times (\lambda_{i}(t)+u_{i\cdot }(t))}{h_{i}\times m_{i}^{t}\times (\lambda_{i}(t)+u_{i\cdot }(t))+\rho_{i}^{\lambda }(t)},i\in N$$

Consequently, the running state vectors of the VEC system can be defined as Equation (6).

(6) $$X=\left[\begin{array}{l} x_{1}\\ \vdots \\ x_{i}\\ \vdots \\ x_{n} \end{array}\right],i\in N$$

Secondly, the running state equation of the VEC system is used to describe the relationship between the input of this system and the system state. The running state equation of this system can be obtained, as shown in Equation (7).

(7) $$\dot{x}=\frac{dX}{dt}$$

Furthermore, Formula (7) can be equivalently transformed into Formula (8).

(8) $$\dot{x}=A(t)x(t)+B(t)u(t)$$

where the matrix A(t) represents the relationship among the state variables within the VEC system. The matrix B(t) represents the state control variable, which is used to control and track the variations of running states.

The CPU utilization over time can be represented by the vector x(t). The vector x(t) is referred as the trajectory of the VEC system. The scheduled tasks among the VEC servers can be denoted as u(t), namely the control vector. Through scheduling tasks among VEC servers, we can effectively control the CPU utilization of VEC servers from the initial running state to the final running state. In the entire running process, the offloaded task execution time and migration time can be controlled in an optimum range during the convergence cycle. Then, the convergence cycle of the VEC system can be denoted as a functional variable J. If we get the minimum value of the vector f by using control vector u(t) to control vector x(t) from the original state x(0) to the destination state $x(t_{f})$, we could get an optimal routing trajectory for all offloaded tasks in the VEC system. Here, the optimal routing trajectory is denoted as $x^{*}(t)$. $t_{f}$ is the time consumption for the optimization process. The corresponding control vector is called optimal control vector, which is denoted as $u^{*}(t)$.

Next, the status information of the servers, such as the CPU utilization and the network bandwidth, is collected by the SDN controller. The offloaded task among these servers is totally scheduled through the designed load-balancing app on a SDN controller. Optimized task scheduling among VEC servers can improve the load imbalance for the VEC virtual resources. Specifically, the VEC servers with higher load will migrate their tasks to that with lower load. Furthermore, the CPU utilization of all the optimized VEC servers will keep on optimum status level.

Finally, a threshold value $C_{\max}\%$ for CPU utilization is pre-defined. When the CPU utilization of a VEC server has just exceeded the pre-defined value, this time is set as the initial state indexed by 0. In addition, the CPU utilization of a VEC server is assumed as the optimization object. After time T, we can obtain the final optimization state. Hence, we can the optimal range for all of the VEC servers between the initial state and the final optimization state through tasks scheduling. Here, the optimum range of CPU utilization for a VEC server is denoted as φ(x(T),T)=0, and the vector form of φ(x(T),T)=0 is represented as G(x(t))=0 and the range of x(T) is set as S={x(T)|G(x(t))=0}.

At last, the proposed Algorithm 1 is shown as below.
**Algorithm 1**. OCTS**Input:** Task arrival numbers, CPU usage of all SBSs, Control parameter ξ, J(u(t)) **Output:** Optimal control $u^{*}(t)$, optimal trajectory $x^{*}(t)$ 1.  **For**
t=0; t≤T
**do** 2.     Calculate the expected CPU usage of SBSs $C_{e}\%$; 3.     Calculate the fitted curve λ(t) and $\rho^{\lambda }(t)$ based on task arrival numbers; 4.     Establish the state vector $\dot{x}$ based on step 2 and 3; 5.     Solving the extreme value of J(u(t)); 6.     Get $u^{*}(t)$ and $x^{*}(t)$**;** 7.     Update the CPU usage of SBSs; 8.  **End For** 9.  **Return**
$u_{1}^{*}(t),$
$\cdots ,u_{t}^{*}(t),$
⋯
$,u_{T}^{*}(t)$

## 6. Experiments

### 6.1. Experiment Setup

In this section, the simulation experiments are designed and implemented to evaluate the performance for the proposed optimal control-based computation task scheduling in software-defined vehicular edge networking in terms of CPU utilization and delay. The minimum configuration of a server is Core i3 CPU and 4G memory for performing the experiment in real time.

**Figure 5:**
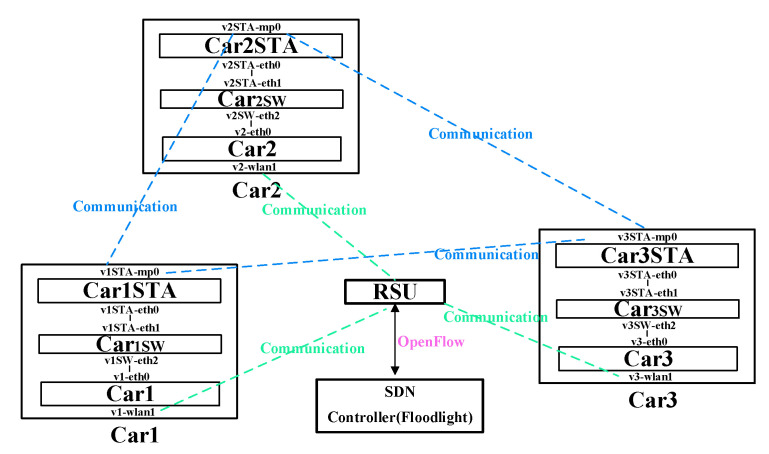
The communication architecture for SD-VEC.

We have presented the software-defined vehicular edge networking (SD-VEC) environments, as shown in Figure 5. As shown in this figure, the vehicle nodes firstly communicate with its RSU node, and then the RSU node can obtain the flow tables through the RSU controller, the vehicle nodes can route by the flow rules at last. Here, we choose the floodlight v1.2 as the SDN controller. The combination of simulation of urban mobility (SUMO v1.8) with instant virtual network (Mininet v2.2) as the base software platform are installed on a computer equipped with i7 CPU and 8GB memory. SUMO can generate the mobility pattern of the vehicles that is used by the Mininet. We chose the city of Luxembourg in European cities as the simulation scenario. That is because Luxembourg SUMO Traffic Scenario is well-known and frequently used to evaluate the VANETs communication system. In this network experiment, there is an SDN controller, there are 5 VEC servers and 100 vehicles in a SD-VEC network. As shown in Figure 5, one VEC server can connect 20 vehicles at the beginning. To simplify the calculation, the computation capacity of every VEC server is set to 1GHz and the task transmission time in the LAN is set to 10 ms without network congestion. The event of vehicular task arrivals on a VEC server follows the Poisson distribution, and every task will take $10^{6}$ CPU cycles. The captured network traffic in this paper is the time series with white Gaussian noise. The rest of simulation experimental parameters are shown in Table 2.

### 6.2. Performance Evaluation

**Figure 6:**
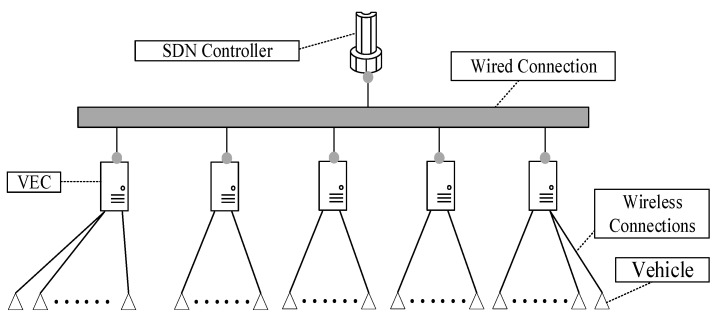
Network topology of the experiment.

Figure 6 shows the network topology of the experiment. In the figure, the SDN Controller is connected with VEC by these cables, and the VECs is connected with the vehicles by wireless.

**Figure 7:**
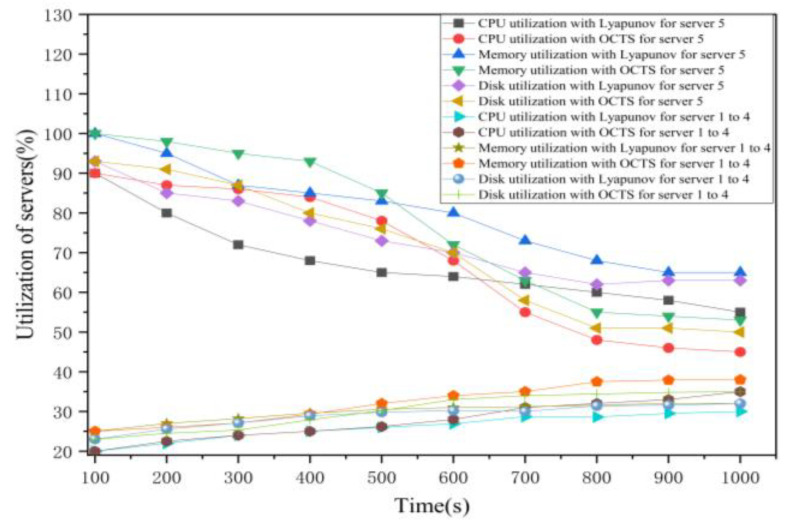
The edge server usage comparisons between Lyapunov optimization and our proposed method.

As shown in Figure 7, the initial average CPU utilization of the edge servers 1 to 4 is 20% and CPU utilization of the edge server 5 is 90%. The initial average memory utilization of the edge servers 1 to 4 is 25% and memory utilization of the edge server 5 is 100%. The initial average disk utilization of the edge servers 1 to 4 is 23% and memory utilization of the edge server 5 is 93%. The total experiment duration is 1000 s. The experiments show that the usages of network load are uneven. Next, we simulated the Lyapunov optimization algorithm to improve the efficiency of the task scheduling on the edge servers. The average CPU utilization of servers 1 to 4 have increased from 20% to 30% and server 5 has decreased from 90% to 55%. The measurements of CPU utilization in this figure show the CPU utilization curves of the five edge servers are closer than that of without using any optimization. These results show the Lyapunov optimization algorithm can achieve the goal of computation load balancing. Then, we used the proposed OCTS optimization algorithm to schedule the offloaded task on the edge servers. As shown in Figure 7, the CPU utilization of servers 1 to 4 have increased from 20% to 35%, and the CPU usage of server 5 has decreased from 90% to 45%. The measurements of CPU utilization in Figure 7 show the CPU utilization curves of the five edge servers are closer than that of no optimization and using Lyapunov optimization. These experimental results prove that our proposed OCTS method is better than the Lyapunov optimization and no optimization methods in terms of computation load balancing. With the Lyapunov optimization, the average memory utilization of servers 1 to 4 have increased from 25% to 32% and server 5 has decreased from 100% to 65% while with the OCTS optimization, the average memory utilization of servers 1 to 4 have increased from 25% to 38% and server 5 has decreased from 100% to 53%. With the Lyapunov optimization, the average disk utilization of servers 1 to 4 have increased from 23% to 32% and server 5 has decreased from 93% to 63% while with the OCTS optimization, the average memory utilization of servers 1 to 4 have increased from 23% to 35% and server 5 has decreased from 93% to 50%.

As shown in Figure 7, in the early stage of optimization, the effect of OCTS is not as good as that of Lyapunov. This is because if too many vehicular tasks are migrated at that time, there will be a high migration delay. When the load of the server drops, the optimization effect of OCTS is obviously better than that of Lyapunov, because when the computing delay of the server drops, the migration of a large number of vehicular tasks will not lead to too high migration delay.

Hence, we can get a conclusion that the optimization effect for the proposed OCTS algorithm is about 10% to 15% higher than the Lyapunov optimization method under the same status conditions. Furthermore, the optimization effect for the proposed OCTS algorithm is about 30% to 40% higher than no optimization method under the same status conditions.

**Figure 8:**
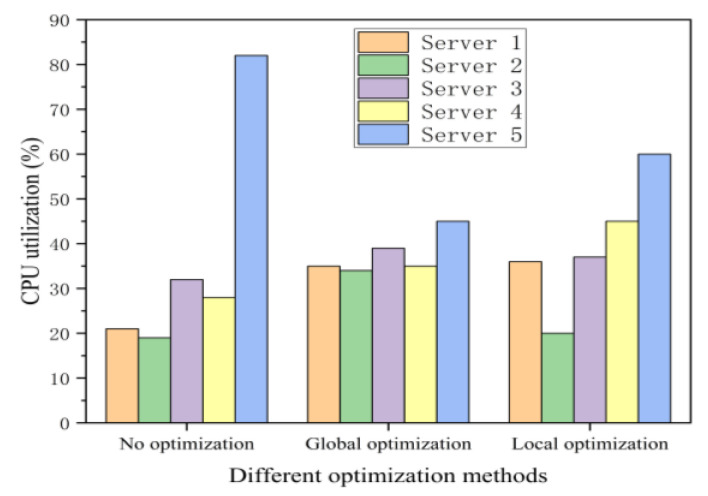
CPU utilization comparisons among the local optimization, no optimization, and global optimization methods.

Secondly, we compare the CPU utilization among the no optimization, the local optimization, and global optimization, as shown in Figure 8. Global optimization means that we can get load and delay information for each server through the SDN controller. In this figure, when the system runs without optimization, server 2 is about 20% CPU utilization and server 4 is about 30% CPU utilization. When the system is under local optimization, server 2 is about 20% CPU utilization and server 4 is about 45% CPU utilization. At this time, server 5 offloads a large number of tasks to server 4, and server 2 remains idle due to the lack of global performance status information. Tasks from server 5 is denied when server 4’s CPU utilization is approaching the threshold.

Hence, when the system is under the global optimization, the CPU utilization of server 2 is about 33%, and that of server 4 is about 34%. The experimental results show that our proposed algorithm can achieve more excellent load balancing effect than another two methods.

**Figure 9:**
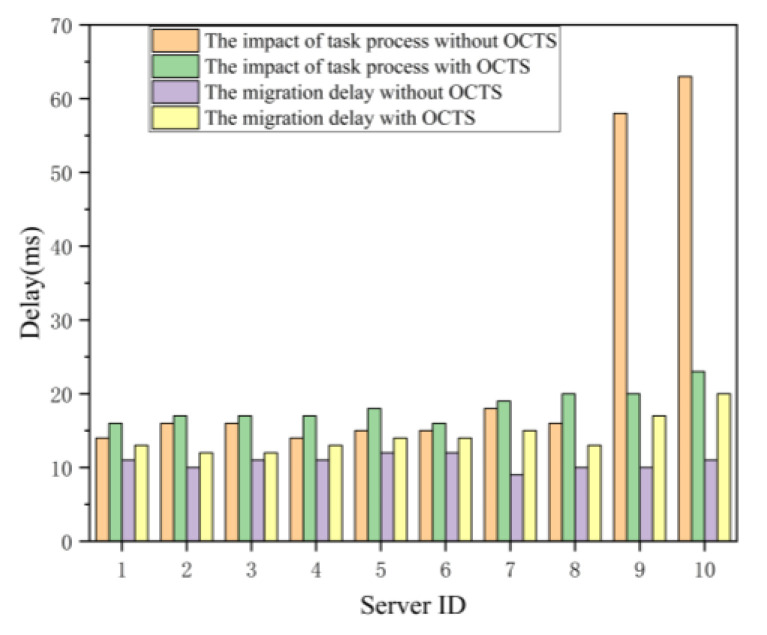
Delay variations on individual servers.

Thirdly, we compare the no optimization with the OCTS optimization methods in the terms of the delay metric in unit of millisecond. As shown in Figure 9, the proposed OCTS method is compared with no optimization method in terms of both the task migration time and the task processing time. The experimental results show that both the task processing and task migration time for the servers 1 to 8 slightly increases when our proposed OCTS method enables. Besides, although the task migration time for servers 9 and 10 have also slightly increases, the task processing time of server 9 and 10 has obviously decreased. After the OCTS optimization finished, the task processing time and the task migration time among the edge servers have become approximately equal. The experimental results prove that our proposed OCTS method can obviously improve the computation and network load balancing. However, the task migration time needs to further optimize. In this evaluation, we set the parameter ξ equal to 1. Next, we try to adjust the value of parameter ξ to optimize the solving. The parameter ξ is used to adjust the proportion of network delay in the load balancing optimization process for reducing the task migration delay. That is because the improvement of load balancing needs too large a number of task migrations from one edge server to another edge server.

**Figure 10:**
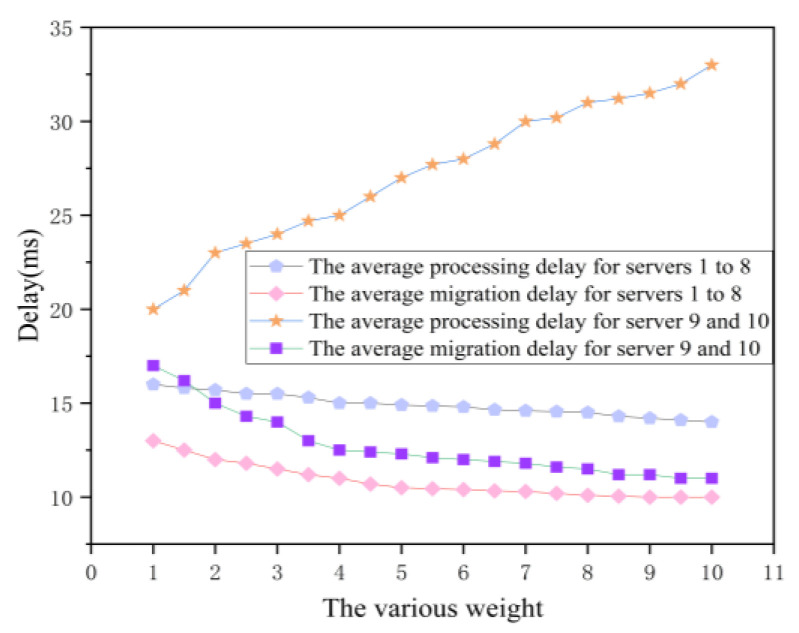
The impact on delay for servers 1 to 10.

Fourthly, we study the parameter ξ optimization to reduce the task migration time. Here, we set a counter for an edge server to count the number of vehicular tasks arriving at the edge server. When the number of the offloaded task is up to 100, the counter will be reset to 0. Besides, the task execution time and task migration time will be calculated again. Then, we try to increase the value of parameter ξ to show the variations of task execution time and task migration time. As shown in Figure 10, compared with no optimization method, the experimental results show that both the task processing time and the task migration time of servers 1 to 8 decreases with the increasing of the value of ξ when our proposed OCTS method enables. Additionally, the task execution time of servers 9 and 10 are significantly reduced after the OCTS optimization. That is because a large number of tasks are scheduled from edge servers 9 and 10 to other edge servers. Meanwhile, the task migration time is doubled. That is because with the increasing value of parameter ξ, the OCTS optimization method appears over fit. As the experimental results show, we can come to the conclusion that there exists an optimal tradeoff between task execution time and task migration time when ξ is equal to 3.

## 7. Conclusions

In this paper, we propose an optimal control-based resource allocation algorithm, called OCTS, and exploit the software-defined networks framework to achieve the computation and network load balancing among the VEC servers in vehicular edge networking. Our contributions are that ① SD-VEC networking layer architecture is presented; ② the calculus of variations method in modern control theory is introduced to solve the optimal load balancing strategy for the offloaded edge computation tasks; ③ the parameter adjustment can optimize the task processing time and task migration time and meet the service requirements of vehicular users. Simulation experimental results prove that our proposed OCTS method can effectively reduce the load imbalance and improve the resource utilization of idle edge servers. Our future work is to study the user behavior and predict the load imbalance position in advance. According to the load utilization estimation, we can effectively schedule the vehicular tasks to the idle VEC servers.

## Figures and Tables

**Table 1:** Description of the symbols.

Symbols	Description
$f_{i}$	The CPU frequency of VEC server i
$h_{i}$	The CPU clock period of VEC server i
$\lambda_{i}(t)$	The number of the tasks arriving at VEC server i at time t
$m_{i}^{t}$	The task execution time on server i at time t
$\rho_{i}^{\lambda }$	The processing time to run tasks on server i
$u_{ij}(t)$	The number of the tasks sent form server i to server j
τ	The task transmission time with no network congestion
$ND_{i}$	The task transmission time on server i
$CD_{i}$	The task execution time on server i
$x_{i}$	State variable for VEC server i
J	The convergence cycle of the system at time t
$x^{*}(t)$	The optimal routing trajectory at time t
$u^{*}(t)$	The optimal control vector at time t
$C_{\max}$	Threshold value of CPU utilization
φ	The optimum range of CPU utilization for a VEC server
G	The vector form of φ
D	The execution time and transmission time incurred by offloaded task scheduling
ξ	A coefficient which is used to adjust the weight of the offloaded task transmission time

**Table 2:** Description of the simulation experimental parameters.

Impact Factors on CPU Utilization	CPU Utilization of Servers 1 to 5				
	1	2	3	4	5
The first set of servers without any impact	20	20	30	30	80
The impact of Lyapunov on the first set of servers	33	33	35	35	48
The impact of OCTS on the first set of servers	35	35	37	37	45
The second set of serverswithout any impact	20	20	30	30	90
The impact of Lyapunov on second set of servers	33	33	35	35	53
The impact of OCTS on the second set of servers	35	35	39	39	49

## Data Availability

Not applicable.
